# Cornel Iridoid Glycoside Protects Against STAT1-Dependent Synapse and Memory Deficits by Increasing *N*-Methyl-D-aspartate Receptor Expression in a Tau Transgenic Mice

**DOI:** 10.3389/fnagi.2021.671206

**Published:** 2021-05-25

**Authors:** Denglei Ma, Rui Huang, Kaiwen Guo, Zirun Zhao, Weipeng Wei, Lihong Gu, Lin Li, Lan Zhang

**Affiliations:** ^1^Key Laboratory for Neurodegenerative Diseases of Ministry of Education, Department of Pharmacy, Beijing Institute for Brain Disorders, Beijing Engineering Research Center for Nerve System Drugs, National Clinical Research Center for Geriatric Diseases, Xuanwu Hospital of Capital Medical University, Beijing, China; ^2^Renaissance School of Medicine at Stony Brook University, Stony Brook, NY, United States

**Keywords:** tau accumulation, cornel iridoid glycoside, P301S transgenic mouse, Alzheimer’s disease, Janus kinase-2/STAT1, NMDAR, tauopathy

## Abstract

P301S transgenic mice are an animal model of tauopathy and Alzheimer’s disease (AD), exhibiting tau pathology and synaptic dysfunction. Cornel iridoid glycoside (CIG) is an active ingredient extracted from *Cornus officinalis*, a traditional Chinese herb. In the present study, the purpose was to investigate the effects and mechanisms of CIG on tau pathology and synaptic dysfunction using P301S transgenic mice. The results showed that intragastric administration of CIG for 3.5 months improved cognitive impairments and the survival rate of P301S mice. Electrophysiological recordings and transmission electron microscopy study showed that CIG improved synaptic plasticity and increased the ultrastructure and number of synapse. Moreover, CIG increased the expression levels of *N*-methyl-D-aspartate receptors (NMDAR) subunits GluN1, GluN2A, and GluN2B, and α-amino-3-hydroxy-5-methyl-4-isoxazole propionic acid receptor (AMPAR) subunit GluA1. We inferred that the major mechanism of CIG involving in the regulation of synaptic dysfunctions was inhibiting the activation of Janus kinase-2 (JAK2)/signal transducer and activator of transcription 1 (STAT1) signaling pathway and alleviating STAT1-induced suppression of NMDAR expressions. Based on our findings, we thought CIG might be a promising candidate for the therapy of tauopathy such as AD.

## Introduction

Alzheimer’s disease (AD) is a common neurodegenerative disease characterized by progressive dementia and results in an enormous emotional and financial burden in the patients and society worldwide (Jia et al., 2020). Under normal conditions, microtubule-associated protein tau functions to regulate microtubules assembly and stability. However, hyperphosphorylated tau tends to accumulate into oligomers, paired helical filaments (PHFs), and eventually intracellular neurofibrillary tangles (NFTs), which are toxic to the synapses and neurons and lead to the impaired cognitive impairments in AD (Gong and Iqbal, 2008). Besides AD, hyperphosphorylated and pathological tau is also a character of tauopathies, a series of diseases including corticobasal degeneration, frontotemporal dementia with parkinsonism-17, and Pick’s disease (Šimić et al., 2016). Rescue of synaptic and neuronal connectivity loss by inhibition of tau pathology is reported as the most promising treatment for tauopathies, such as AD (Iqbal et al., 2018).

Many studies have demonstrated the existence of hyperphosphorylated tau formulated oligomers in the brain of patients and transgenic animal models (Ren and Sahara, 2013). P301S transgenic mouse PS19 strain was generated by Yoshiyama et al. (2007). Filamentous tau lesions began to develop in P301S mice since 6 months of age and progressively accumulated in association with neuronal loss at 9–12 months of age (Yoshiyama et al., 2007). Remarkably, hippocampal synapse loss and impaired synaptic functions were detected in 3-month-old P301S mice before NFTs emerged (Yoshiyama et al., 2007). *In vivo* measurement of glutamate loss is reported to be in association with synapse loss and synaptic dysfunction in P301S mice (Crescenzi et al., 2014). These results showed that synaptic pathology might be one of the earliest neurotoxic consequences of pathogenic human tau expression in animal models of tauopathy.

Cornel iridoid glycoside (CIG) is the active ingredient extracted from *Cornus officinalis*, which is a widely known traditional Chinese herb used for treating age-related neurodegenerative diseases and dementia. In our previous study, we found that CIG ameliorated cognitive functions, increased neuronal survival and expression of neurotrophic factors in the rat brain post traumatic injury, and inhibited tau hyperphosphorylation in the brain of senescence-accelerated mouse P8 (SAMP8) (Ma et al., 2016, 2018). Our studies also showed that CIG inhibited tau hyperphosphorylation and pathology by regulating protein phosphatase 2A (PP2A) both *in vivo* and *in vitro* (Yang et al., 2013; Ma et al., 2019). Moreover, we recently found that CIG alleviated the synaptic dysfunctions in tau transgenic rTg4510 mice (Ma et al., 2020). However, the mechanism by which CIG improves synaptic dysfunction in tauopathy remains unclear. In the present study, we applied P301S transgenic mice to investigate the pharmacological effects and underlying mechanisms of CIG on tau pathology and synaptic functions.

## Materials and Methods

### Drugs

Cornel iridoid glycoside was extracted from the sarcocarp of *Cornus officinalis* Sieb. et Zucc, as described in our previous article (Yao et al., 2009). The main active compounds of CIG were morroniside and loganin, in which morroniside accounted for 67% and loganin 33%, and the remaining 30% contain some small peaks, which were unable to be separated, purified, and identified. Memantine, as the positive control drug in this study, was purchased from H. Lundbeck A/S (Lot No. 47195). Recent studies found that memantine could inhibit tau hyperphosphorylation and pathology as an approved drug for AD (Folch et al., 2018).

### Animals and Their Assigned Groups

P301S transgenic mice of PS19 strain (male and female in half) were generated as previously reported by the Nanjing Biomedical Research Institute of Nanjing University, China (Yoshiyama et al., 2007). All mice were housed under standard temperature conditions and a light–dark cycle (12:12), with free access to food and water. All animal care and experimental procedures were performed according to the requirements of the provisions and general recommendations of Chinese Experimental Animal Administration Legislation and the National Institutes of Health guide for the care and use of laboratory animals. The present study was approved by the Bioethics Committee of Xuanwu Hospital of Capital Medical University.

P301S transgenic mice were randomly assigned to P301S Tg group (treated with saline), three groups of three-dose CIG treatment P301S mice (50, 100, and 200 mg/kg, respectively), and memantine-treated P301S mice group (5 mg/kg); *n* = 20 for each group. Forty non-transgenic littermates were randomly assigned to nTg control group and nTg mice treated with CIG (100 mg/kg) group; *n* = 20 per group. Drugs were dissolved in saline and intragastrically administered once a day for 3.5 months from 9 to 12.5 months of age. Control and model groups intragastrically received an equal volume of saline.

### Objective Recognition Test

A white plastic chamber was located in a quiet room dimly with illumination of 40 lx. On Day 1, mice were allowed to adapt to the chamber for 5 min. On Day 2, they were re-introduced in the chamber for 5-min exploration with two clean plastic objects located at two opposite sides of the arena. On Day 3, one of the objects was replaced with a new object, and mice were re-placed in the chamber to start a 5-min object recognition test. Cumulative time spent in object exploration was recorded and used to calculate a memory discrimination index (DI): DI = (N − F)/(N + F), where N is the elapsed time of exploring the new object and F is the time spent exploring the familiar object. A higher DI value represented better memory ability.

### Y-Maze Test

The Y maze consists of three identical arms, each of which is 30 cm × 10 cm × 25 cm (length × width × height). The angle of each arm was 120°. Arms are labeled A, B, and C. The apparatus was placed 40 cm above the floor. Each mouse was placed at the end of one arm and allowed to move freely through the maze during an 8-min session. The arm entry sequences were recorded (e.g., ABCBAC). A series of entries into all three arms on consecutive occasions were defined as an actual alternation. The maximum alternation was the total number of arm entries minus two. The percentage of alternation was calculated using the following formula: (actual alternations/maximum alternations) × 100% (Sun et al., 2017).

### Hippocampal Slice Recordings

Procedures for hippocampus slice preparation and electrophysiological recordings followed a well-established protocol (Takeuchi et al., 2015; Gelman et al., 2017). Brains were rapidly removed from anesthetized mice and submerged in cold artificial cerebrospinal fluid (ACSF) continuously bubbled with 95% O_2_/5% CO_2_. The ACSF composition in mM was as follows: 124 NaCl, 2.5 KCl, 1.2 NaH_2_PO_4_, 24 NaHCO_3_, 5 HEPES, 12.5 D-glucose, 2.0 CaCl_2_, and 1.5 MgSO_4_. Transverse slices (380 μm thick) containing hippocampus were prepared using a vibrating blade microtome (Leica, VT 1,000 S, Germany) and pre-incubated in an incubation chamber with oxygenated ACSF at room temperature for at least 1 h before use. Slices were transferred to an interface chamber that was continually superfused with ACSF at a rate of 3 ml/min. Slices were maintained at 32 ± 1.5°C while recording. The field excitatory postsynaptic potentials (fEPSPs) were evoked by a constant stimulation in the Schaffer collaterals with a microelectrode (glass microelectrode filled with ACSF with resistance of 3–4 MΩ) and recorded in the stratum radiatum layer of CA1 with a bipolar electrode filled with ACSF. The stimulus intensity was set to evoke about 40% of the maximal amplitude of fEPSPs. After the baseline measurements were recorded, long-term potentiation (LTP) was induced by high-frequency stimulation (three 1-s, 100-Hz stimulus trains delivered 10 s apart), and responses were recorded for 60 min after the stimulation using a Axopatch 700B amplifier and a Digidata 1440A data acquisition device (Axon Instruments, CA, United States) controlled by pClamp10.5 software. The normalized LTPs are quantified as the mean percentage of baseline fEPSP slope by Clampfit10 software.

### Transmission Electron Microscopy

After behavioral testing, three mice in each group were anesthetized and perfused with 2.5% glutaraldehyde. The brains were harvested, and small brain block of 1 mm^3^ were cut from the hippocampus and fixed with 2.5% glutaraldehyde for 4-6 h and in 1% osmium tetroxide for 2 h at 4°C. Ultrathin sections were prepared by ethanol dehydration and epoxy resin embedding and then stained with uranyl acetate solution and lead citrate solution. After rinsing, the ultrastructure of the synapse was observed using a JEM 2100 transmission electron microscope (Nissan Chemical Industry Co., Ltd., Tokyo, Japan) and photographed. From each section, 10 fields of vision were randomly photographed at a magnification of 4,000 × for counting the number of synapses.

### Immunohistochemical Staining of AT8

For histochemical analysis of the brain, mice were anesthetized and perfused transcardially with 0.01 M of phosphate-buffered saline (PBS) (pH 7.4) and 4% paraformaldehyde in 0.01 M of PBS. The brain was removed and then switched to 30% sucrose/0.1 M of PBS. After being frozen in isopentane, the brain tissues were cut in serial, 30-μm-thick horizontal sections in a cryostat slicer (CM1900, Leica, Germany). The brain sections were exposed to 3% H_2_O_2_ for 10 min, blocked by 5% normal goat serum in PBS for 60 min, and then incubated with AT8 (1:200, an antibody against PHF tau phosphorylated at Ser202/Thr205; Thermo Fisher Scientific, United States) at 4°C overnight. The sections were incubated with a goat anti-mouse non-biotin detection system (Zsbio, China) at 37°C and visualized using 3,3′-diaminobenzidine (DAB) substrate kit (Zsbio, China). Sections were counterstained with hematoxylin, dehydrated in ascending concentrations of ethanol, and coverslipped. Slices were imaged using the Olympus microscope (BX51, Olympus, Japan) and Pixera TWAIN View Finder Pro system. Images were quantified using IHC Profiler and threshold in ImageJ software.

### Western Blotting

The brain tissue of four mice in each group was homogenized in ice-cold lysis buffer containing 50 mM of Tris–HCl (pH 7.4), 150 mM of NaCl, 2 mM of EGTA, 1% Non-idet P-40, 0.1% sodium dodecyl sulfate (SDS), 0.5% sodium deoxycholate, phosphatase, and protease inhibitor cocktail (Merck Millipore, Germany). Homogenates were centrifuged at 12,000 × *g* for 20 min at 4°C, and supernatants were collected and stored at −80°C. Consequently, protein concentration was determined using a BCA Protein Assay Kit (Thermo Fisher Scientific, United States).

Proteins were separated on SDS–Tris-glycine polyacrylamide gel and transferred to polyvinylidene difluoride (PVDF) membrane. The membranes were then blocked with 5% non-fat milk in Tris-buffered saline–Tween 20 consisting of 10 mM of Tris–HCl, 100 mM of NaCl, and 0.05% Tween-20 and incubated with primary antibodies at 4°C overnight. The primary antibodies used in this study were tau5 (cat. no. 577801, anti-total tau, 1:1,000, Merck Millipore, Germany), T22 (cat. no. ABN454, anti-tau oligomers, 1:1,000, Merck Millipore, Germany); anti-phosphorylated (p)-tau at Ser396 (pS396, cat. no. 44-752G, 1:1,000, Thermo, United States); anti-GluN1 (cat. no. ab193310), anti-GluN2A (cat. no. ab169873), anti-GluN2B (cat. no. ab65783), anti-GluA1 (cat. no. ab31232), anti-GluA2 (cat. no. ab20673), anti-signal transducer and activator of transcription 1 (STAT1, cat. no. ab155933, 1:1,000), and anti-phosphorylated STAT1 (Tyr701, cat. no. ab29045) purchased from Abcam, United States; anti-Janus kinase-2 (JAK2, cat. no. 3230), anti-phosphorylated JAK2 (Tyr1007/1008, cat. no. 3771) and anti-GAPDH antibodies (cat. no. 5174, 1:1,000, Cell Signaling Technology, United States). After incubation with horseradish peroxidase-conjugated anti-rabbit or anti-mouse IgG antibody (1:2,000) for 1 h, immune complex was detected by enhanced chemiluminescence (ECL) detection reagents (Merck Millipore, Germany). Intensity of the bands on membranes was analyzed using Multi Gauge V3.0 (Fuji Pharma, Japan).

### Statistical Analysis

All data were analyzed and plotted using Prism version 5.0 software (GraphPad Software Inc., United States) and one-way ANOVA followed by a Dunnett’s *post hoc* test to determine statistical significance among groups. Surviving rate was analyzed using Kaplan–Meier analysis. Numerical data were provided as mean ± standard error of the mean (SEM), and *P* value less than 0.05 was considered to be statistically significant.

## Results

### Cornel Iridoid Glycoside Improves Cognitive Impairment and Survival Rate of P301S Transgenic Mice

**FIGURE 1:**
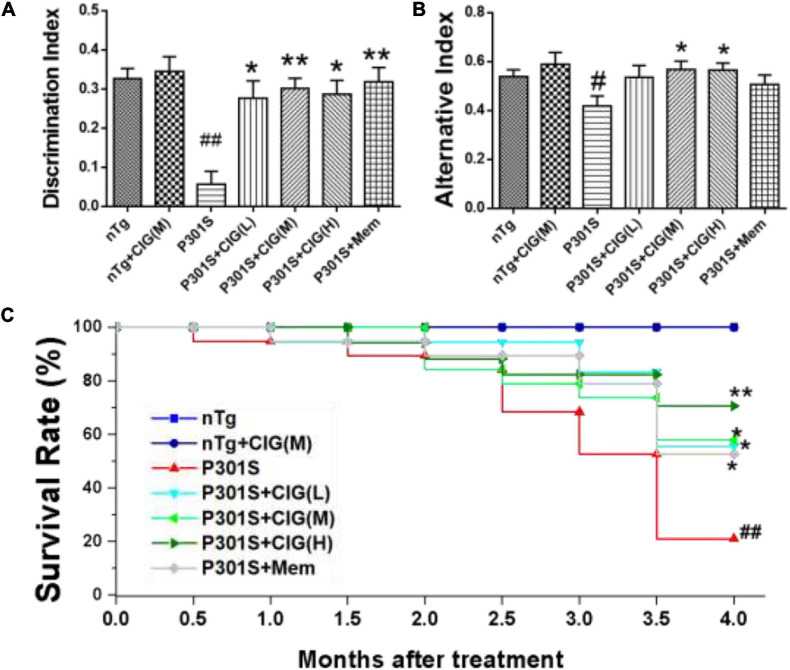
Cornel iridoid glycoside (CIG) improved cognitive impairments and survival rate of P301S transgenic mice. CIG was intragastrically administered to P301S mice for 3.5 months. **(A)** Discrimination index (DI) in object recognition test. **(B)** Percentage of alternation behavior (alternation index) in the Y-maze test. **(C)** Survival rate of 9-month-old P301S mice after treatment. Data are expressed as mean ± SEM; *n* = 12–20 each group. ^#^*P* < 0.05, ^##^*P* < 0.01, P301S transgenic mice *vs* nTg control mice; *P < 0.05, **P < 0.01, drug-treated P301S mice *vs* saline-treated P301S mice. nTg, non-transgenic control mice; nTg + CIG(M), non-transgenic control mice treated with CIG (100 mg/kg, medium dose); P301S, vehicle-treated P301S mice; P301S + CIG(L), CIG (50 mg/kg, low dose)-treated P301S mice; P301S + CIG(M), CIG (100 mg/kg, medium dose)-treated P301S mice; P301S + CIG(H), CIG (200 mg/kg, high dose)-treated P301S mice; P301S + Mem, P301S mice treated with 5 mg/kg of memantine.

Object recognition test indicates recognition cognitive ability of P301S transgenic mice. The results showed that DI in P301S Tg mice was evidently lower than that in nTg mice (*P* < 0.01); CIG (50, 100, and 200 mg/kg) treatment in P301S mice significantly elevated DI in P301S mice (*P* < 0.05, *P* < 0.01; Figure 1A). Spontaneous alternation behavior in Y maze was performed to evaluate the spatial learning and memory abilities of mice. The present study showed that the percentages of alternation behavior in Y maze of P301S Tg mice were remarkably lower than those in nTg mice (*P* < 0.05; Figure 1B), and CIG treatment at 100 and 200 mg/kg evidently increased the percentages of alternation behavior of P301S mice (*P* < 0.05; Figure 1B). These results indicated that CIG ameliorated the non-spatial and spatial cognitive impairments in P301S Tg mice.

P301S mice have shortened survival time due to tauopathy and hindlimb paralysis (Yoshiyama et al., 2007). During the period from 9 to 13 months of age, many P301S mice died, showing a more sharper surviving rate than nTg mice (*P* < 0.01; Figure 1C). P301S mice treated with CIG showed higher surviving rate than P301S model group (*P* < 0.05, *P* < 0.05; Figure 1C). These results indicated that CIG increased the survival rate and prolonged the life span of P301S mice.

### Cornel Iridoid Glycoside Protects Synaptic Plasticity and Synapse Ultrastructure in the Hippocampus of P301S Mice

**FIGURE 2:**
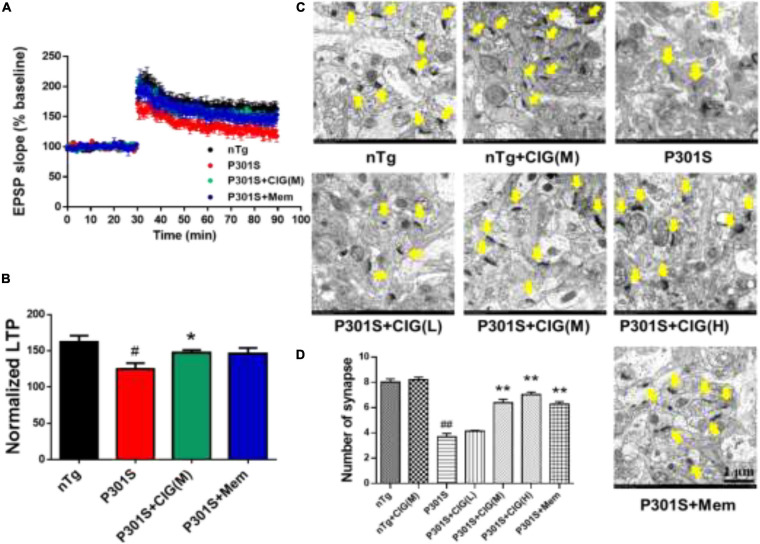
Cornel iridoid glycoside (CIG) protects synaptic plasticity and synapse ultrastructure in the hippocampus of P301S mice. **(A)** Long-term potentiation (LTP) was induced by high-frequency stimulus trains after 30-min baseline stimulation. The field excitatory postsynaptic potentials (fEPSPs) were recorded for 60 min. **(B)** Statistical analysis of average LTP responses between 0 and 60 min after high-frequency stimulus trains. **(C)** Representative images of the ultrastructure of synapses in the hippocampus of mice detected by transmission electron microscopy (yellow arrow). Magnification: 4,000 × scale bar = 1 μm. **(D)** Quantitative analysis of the number of synapses in the hippocampus of mice. Data are expressed as mean ± SEM; *n* = 6 each group. ^#^*P* < 0.05, ^##^*P* < 0.01, P301S transgenic mice *vs* nTg control mice; *P < 0.05, **P < 0.01, drug-treated P301S mice *vs* saline-treated P301S mice. nTg, non-transgenic control mice; P301S, P301S transgenic mice; CIG(L), CIG 50 mg/kg, low dose; CIG(M), CIG 100 mg/kg, medium dose; CIG(H), CIG 200 mg/kg, high dose; Mem, memantine 5 mg/kg.

Synaptic dysfunction is an early feature of AD and is believed to be the basis of cognitive impairment. Synaptic plasticity plays an important role in learning and memory, which can be measured by LTP. Brain slices from P301S mice showed lower fEPSP slope, indicating less responsiveness to stimulation than those from nTg mice, and CIG treatment increased fEPSP slope of P301S mice (Figure 2A). The normalized LTP at the 60 min after stimulation in P301S mice group was significantly decreased (*P* < 0.05), and CIG treatment at 100 mg/kg evidently elevated the normalized LTP (*P* < 0.05, Figure 2B).

To investigate the effect of CIG on the synaptic loss, we assessed the synaptic ultrastructure and density in the CA1 area by transmission electron microscopy. Compared with nTg mice, P301S mice displayed blurred structures and fewer synapses (*P* < 0.01; Figures 2C,D). CIG treatment protected the synapse ultrastructure and significantly increased the number of synapses in the hippocampus of P301S mice (*P* < 0.01; Figures 2C,D). These results indicated that CIG ameliorated synapse loss and the synaptic plasticity deficits (impaired LTP) in P301S mice.

### Cornel Iridoid Glycoside Increased the Expression of *N*-Methyl-D-aspartate Receptors and α-Amino-3-hydroxy-5-methyl-4-isoxazole Propionic Acid Receptors in the Brain of P301S Mice

**FIGURE 3:**
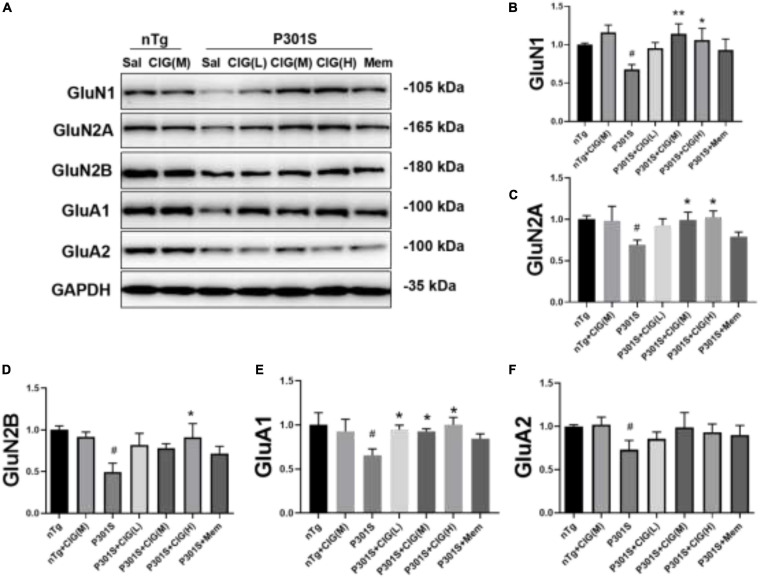
Cornel iridoid glycoside (CIG) increased the expression of NMDARs and AMPARs in the brain of P301S transgenic mice. **(A)** Representative images of Western blotting for NMDAR subunits GluN1, GluN2A, and GluN2B and AMPAR subunits GluA1 and GluA2 in the brain of mice. Quantification of Western blotting for NMDAR subunits GluN1 **(B)**, GluN2A **(C)**, and GluN2B **(D)** and AMPAR subunits GluA1 **(E)** and GluA2 **(F)** in the brain of P301S mice. Quantification of blots after normalization with GAPDH, and the numerical value of nTg group was taken as 1. Data are expressed as mean ± SEM; *n* = 4 each group. ^#^*P* < 0.05, P301S transgenic mice *vs* nTg control mice; *P < 0.05, **P < 0.01, drug-treated P301S mice *vs* saline-treated P301S mice. Sal, saline-treated; NMDARs, *N*-methyl-D-aspartate receptors; AMPARs, α-amino-3-hydroxy-5-methyl-4-isoxazole propionic acid receptors.

On postsynaptic membrane, *N*-methyl-D-aspartate receptor (NMDAR) and α-amino-3-hydroxy-5-methyl-4-isoxazole propionic acid receptor (AMPAR) are closely in association with synaptic plasticity and normal cognitive functions. In the present study, we detected the expression levels of NMDAR subunits GluN1, GluN2A, and GluN2B and AMPAR subunits GluA1 and GluA2 in the brain of P301S mice using Western blotting analysis. The results showed that the expression levels of GluN1, GluN2A, GluN2B, GluA1, and GluA2 significantly declined in the brain of P301S mice (*P* < 0.05; Figure 3); and CIG treatment evidently increased the expressions of GluN1, GluN2A, GluN2B, and GluA1 (*P* < 0.05, *P* < 0.05; Figure 3), suggesting that CIG might protect synapse by increasing the expression of NMDARs and AMPAR GluA1 in the brain of P301S mice.

### Cornel Iridoid Glycoside Inhibited Tau Hyperphosphorylation and Accumulation in the Brain of P301S Mice

**FIGURE 4:**
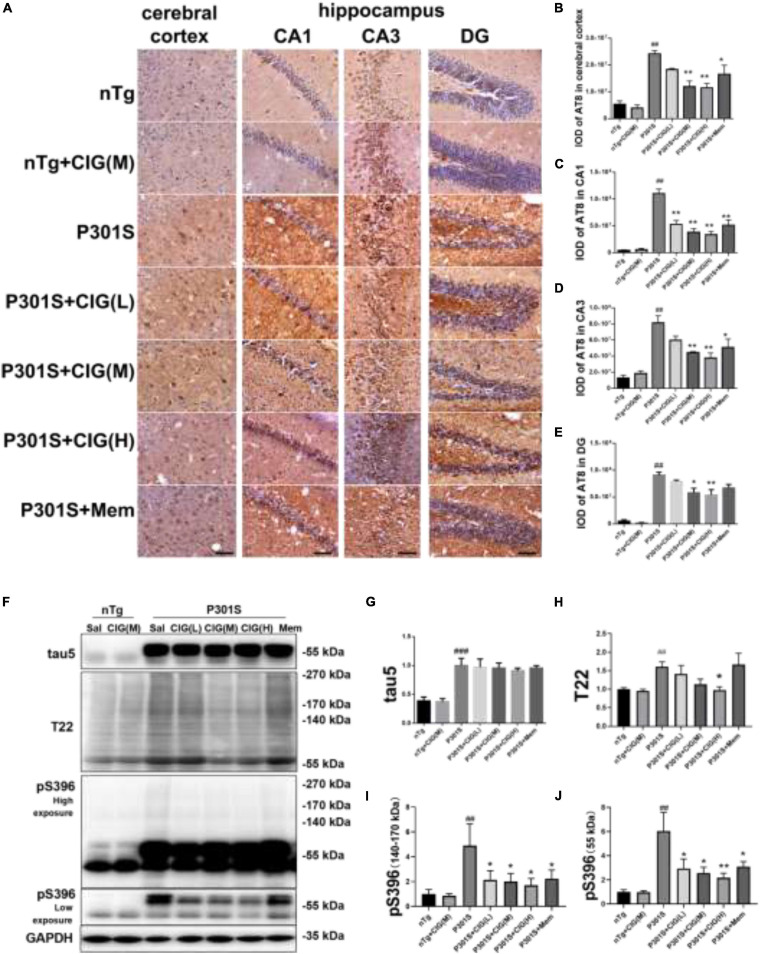
Cornel iridoid glycoside (CIG) inhibited tau phosphorylation and accumulation in the brain of P301S transgenic mice. **(A)** Representative images of immunohistochemical staining of AT8 in the cerebral cortex, and hippocampus CA1, CA3, and DG areas of mice. Scale bar = 50 μm. Quantitative analysis of integrated optical density (IOD) of AT8 in the cerebral cortex **(B)** and hippocampus CA1 **(C)**, CA3 **(D)**, and DG **(E)** areas of mice. **(F)** Representative images of Western blotting for tau5 (total tau), T22 (tau oligomers), monomer (low exposure), and oligomers (high exposure) of phosphorylated (p)-tau at Ser396 (pS396) in the brain of mice. Quantification of Western blotting for tau5 **(G)**, T22 **(H)**, pS396-tau oligomers [140∼170 kDa, **(I)**], and monomer [∼55 kDa, **(J)**]. Quantification of blots after normalization with GAPDH, and the numerical value of nTg group was taken as 1. Data are expressed as mean ± SEM; *n* = 3 per group for immunohistochemical staining; *n* = 4 per group for western blotting. ^##^*P* < 0.01, ^###^*P* < 0.001, P301S transgenic mice *vs* nTg control mice; *P < 0.05, **P < 0.01, drug-treated P301S mice *vs* saline-treated P301S mice.

Immunohistochemical staining of AT8 (against PHF-tau phosphorylated at Ser202/Thr205) indicated tau hyperphosphorylation and aggregation in the cerebral cortex and hippocampus of P301S mice compared with nTg mice (*P* < 0.01); CIG treatment evidently reduced tau hyperphosphorylation and aggregation detected by AT8 staining in the cerebral cortex, hippocampus CA1, CA3, and DG area of P301S mice (*P* < 0.05, *P* < 0.01; Figures 4A–E).

Western blot analysis showed that the expression of total tau (using tau5 antibody), tau oligomers (using T22 antibody), monomer, and oligomers of phosphorylated (p)-tau at Ser396 increased obviously in the brain of P301S mice, compared with the nTg mice (*P* < 0.01, *P* < 0.001; Figures 4F–J). CIG treatment declined the levels of total tau oligomers, monomer (55 kDa) and oligomers of phosphorylated (p)-tau at Ser396 (140∼170 kDa, *P* < 0.05, *P* < 0.01; Figures 4F–J). These results suggest that CIG reduced tau phosphorylation and accumulation in the brain of P301S mice.

### Cornel Iridoid Glycoside Treatment Inhibited the Janus Kinase-2/Signal Transducer and Activator of Transcription 1 Pathway in the Cerebral Cortex of P301S Mice

**FIGURE 5:**
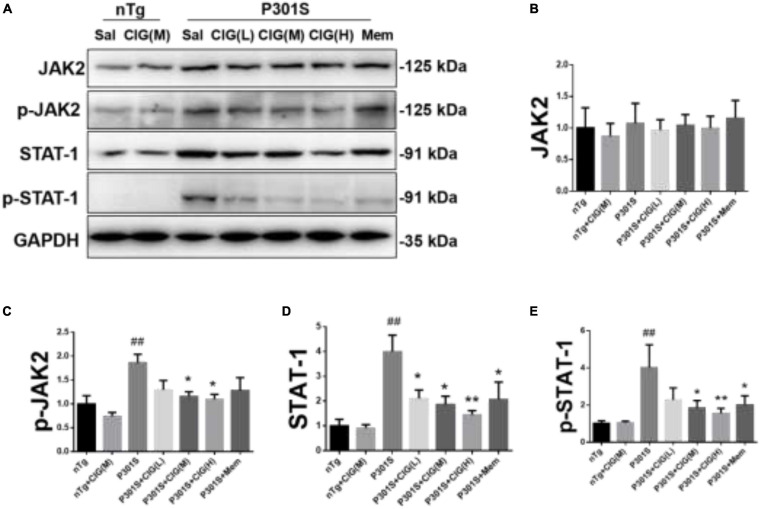
Cornel iridoid glycoside (CIG) treatment inhibited the JAK2/STAT1 signaling pathway in the brain of P301S mice. **(A)** Representative images of Western blotting for JAK2, phosphorylated JAK2 (Tyr1007/1008, p-JAK2), and STAT1, phosphorylated STAT1 (Tyr701, p-STAT1) in the brain of mice. Quantification of Western blotting for JAK2 **(B)**, p-JAK2 **(C)**, STAT1 **(D)**, and p-STAT1 **(E)** in the brain. Data are expressed as mean ± SEM; *n* = 4 each group. ^##^*P* < 0.01, P301S transgenic mice *vs* nTg control mice; *P < 0.05, **P < 0.01, drug-treated P301S mice *vs* saline-treated P301S mice. JAK2, Janus kinase-2; STAT1, signal transducer and activator of transcription 1.

JAK2/STAT1 signaling pathway was reported to be involved with the decreased expression of NMDARs induced by tau pathology (Li et al., 2019). In our present study, we found that the expression level of phosphorylated JAK (p-JAK2, Tyr1007/1008), phosphorylated STAT1 (p-STAT1, Tyr701), and total STAT1 were evidently increased in P301S mice compared with nTg mice (*P* < 0.01; Figure 5). CIG treatment significantly decreased the expression levels of p-JAK2, p-STAT1, and STAT1 (*P* < 0.05, *P* < 0.01; Figure 5). These results indicated that JAK2/STAT1 pathway was activated in the brain of P301S mice, and CIG treatment inhibited the activation of JAK2/STAT1 pathway.

## Discussion

In the present study, intragastric administration of CIG for 3.5 months improved cognitive behaviors in objective recognition test and Y maze task and increased the survival rate of P301S transgenic mice. CIG treatment improved synaptic survival and plasticity, inhibited tau hyperphosporylation and accumulation, increased the expression of NMDARs and AMPARs, and inhibited the activation of JAK2/STAT1 signaling pathway.

Instead of binding to tubulin for microtubule assembly, hyperphosphorylated tau reduces microtubule stabilization, sequesters normal tau from microtubule, and aggregates themselves without inducer molecules (Iqbal et al., 2009). A large body of former studies suggest that tau oligomers may be more cytotoxic than fibrillar aggregates (Lasagna-Reeves et al., 2011; Shafiei et al., 2017). Tau oligomers have been reported to be found in large percentages of synaptic areas in AD patients, suggesting a toxic role of tau oligomers in normal synaptic transmissions (Tai et al., 2014). Several tau transgenic mice models show the structural alterations and electrophysiological changes of synapse (Jadhav et al., 2015). P301S mice begin to show synaptic loss and impaired synaptic function at 3 months of age, which is earlier than the formulation of filamentous tau lesions (Yoshiyama et al., 2007). As tau oligomers are reported to be an early stage in tau pathology in tau Tg mice models, we infer that tau oligomers might be involved in the synaptic dysfunctions in P301S mice.

Recently, the molecular mechanism underlying tau-induced synapse impairment has been discovered by Li et al. (2019). They found that tau accumulation impaired synaptic plasticity through JAK2/STAT1-induced suppression of NMDAR expression. NMDAR and AMPAR belong to ionotropic glutamate receptors and play an important role in synaptic transmission and synaptic plasticity (Zhang et al., 2016). In our previous and present study, we found reduced expression levels of NMDAR and AMPAR in tau Tg mice, including rTg4510 mice (expressing P301L mutant human tau) and P301S mice (expressing P301S mutant human tau) (Ma et al., 2020). And CIG treatment increases the expression of NMDAR and AMPAR in the brain of tau Tg mice, which might partly explain the mechanisms of CIG’s protective effects on synaptic impairments.

The JAK/STAT signaling pathway is reported to be involved in many pathophysiological processes including cell survival, development, and inflammation. JAK2, as a tyrosine kinase, is activated upon intracellular tau accumulation and involved in STAT1 phosphorylation (Quelle et al., 1995; Li et al., 2019). Phosphorylation of STAT1 at Tyr701 is the activated form of this transcription factor, stimulating its activation, dimerization, nuclear translocation, and DNA binding (Liddle et al., 2006). STAT1 can directly bind to the special GAS elements on NMDAR promoters and thus directly block the expression of NMDAR subunits, including GluN1, GluN2A, and GluN2B (Li et al., 2019). In the present study, we found increased expression level of p-JAK2, STAT1, and p-STAT1, indicating the activation of JAK2/STAT1 signaling pathway in the brain of P301S mice. Moreover, we found the decreased expression of GluN1, GluN2A, and GluN2B in the brain of P301S mice, and CIG treatment significantly reversed these changes. Based on these results, we inferred that CIG increased NMDAR expression by inhibiting the accumulation of tau and the activation of JAK2/STAT1 signaling pathway.

In our previous studies, we found CIG showed protective effects on synaptic impairments in several disease models, including vascular dementia, traumatic brain injury, and tauopathy (Ma et al., 2018, 2020; Wang et al., 2020). Furthermore, CIG was reported to inhibit microglia activation through suppression of the JAK/STAT signaling pathway *in vitro* and in an experimental autoimmune encephalomyelitis animal model (Yin et al., 2014; Qu et al., 2019). Tau pathology was reported to be involved in several central nervous system (CNS) diseases, including AD, vascular dementia, traumatic brain injury, and multiple sclerosis (Hu et al., 2018; Chidambaram et al., 2020; Mirzaii-Dizgah et al., 2020; Shi et al., 2020). We inferred that the inhibition of tau accumulation and JAK2/STAT1 pathway activation might underlie the common mechanism of CIG’s pharmacological effects on NMDAR expression and synaptic dysfunction in CNS disorders.

## Conclusion

The current study demonstrated that intragastric administration of CIG for 3.5 months inhibited tau hyperphosphorylation and accumulation into oligomers, increased the expression of NMDARs and AMPARs, improved synaptic plasticity and synapse ultrastructure, and finally resulted in improvement of cognitive functions and survival rate of P301S transgenic mice. The major mechanism of CIG involved in the regulation of synaptic dysfunction was inhibiting the activation of JAK2/STAT1 signaling pathway and STAT1-induced suppression of NMDAR expression. Based on our findings, CIG might be a promising candidate for the prevention of tauopathy such as AD.

## Data Availability Statement

The raw data supporting the conclusions of this article will be made available by the authors, without undue reservation.

## Ethics Statement

The animal study was reviewed and approved by Bioethics Committee of Xuanwu Hospital of Capital Medical University.

## Author Contributions

This study was designed by DM and LZ. Animal experiment was conducted by RH, DM, LG. KG, WW, and ZZ performed other experiments. DM, LL, and LZ analyzed the data and drafted the manuscript. All authors read and approved the final manuscript.

## Conflict of Interest

The authors declare that the research was conducted in the absence of any commercial or financial relationships that could be construed as a potential conflict of interest.
